# The Impact of Range of Motion on Applied Force Characteristics and Electromyographic Activity during Repeated Sets of Bench Press Exercise

**DOI:** 10.5114/jhk/186341

**Published:** 2024-04-15

**Authors:** Athanasios Tsoukos, Michal Wilk, Michał Krzysztofik, Adam Zajac, Gregory C. Bogdanis

**Affiliations:** 1 School of Physical Education and Sports Science, National & Kapodistrian University of Athens, Athens, Greece.; 2 Institute of Sport Sciences, Jerzy Kukuczka Academy of Physical Education in Katowice, Katowice, Poland.

**Keywords:** fatigue, resistance exercise, muscle power, impulse, force plate

## Abstract

This study examined the effects of range of motion (ROM) on applied force, power output and surface electromyographic (sEMG) responses during repeated sets of bench press exercise executed as fast as possible. Ten resistance trained men performed three sets to momentary failure with two-min rest intervals under three different ROM conditions: (a) full ROM (FULL), (b) TOP, at the top half of ROM, and (c) BOTTOM, at the bottom half of ROM. Mean and peak force were higher in TOP compared to FULL and BOTTOM (mean force: 817 ± 80 vs. 657 ± 98 vs. 623 ± 122 N, respectively, p < 0.001) with no differences between FULL and BOTTOM. During repeated sets, large decreases were found in peak (by 29.4 to 45.3%) and mean power (by 55.5 to 64.7%) from the first to the last repetitions. However, the decrease in mean force was only 2% (p < 0.01) and decreases in peak force ranged from 6.7 and 8.8% to zero, indicating the velocity loss was the main contributor to fatigue in power output. Although force and power output in set 3 were unchanged in BOTTOM, mean power output decreased significantly, suggesting that lower performance and fatigue may be related to the longer muscle length. Fatigue was accompanied by an increase in sEMG activity and a decrease in median frequency in all muscles, with triceps brachialis sEMG reflecting more the force and power differences among ROMs. In conclusion, fatigue depends on velocity rather than force loss during bench press exercise at different ROMs.

## Introduction

Range of motion (ROM) has recently been acknowledged as an important variable influencing strength and hypertrophic gains during resistance exercise (Coratella, 2022). The bulk of literature on the impact of ROM on performance and adaptations during resistance exercise compared strength, surface electromyographic (sEMG) responses and fatigue between exercises performed with full and partial ROM (Clark et al., 2008; Cobanoglu et al., 2021; Da Silva et al., 2017; Gillingham et al., 2023; Matykiewicz et al., 2023; Mendonça et al., 2021; Strońska et al., 2018). Depending on whether the partial ROM renders the muscles at longer or shorter length, the ability to exert force may increase or decrease (Butterfield and Herzog, 2005). For example, a previous study found that the 6-repetition maximum (6-RM) load and peak force increased, while the mean concentric work per repetition decreased when the bench press was performed at ¼ ROM from elbow extension compared with full ROM (Clark et al., 2008). Similar findings have been reported for the deadlift exercise, where 1-RM increased by 17.9% and peak and mean power were higher when the exercise was performed for a short part of ROM close to the end of the ROM compared to the full ROM (Drinkwater et al., 2012).

The magnitude of fatigue is also affected by muscle length, and therefore ROM (Bogdanis et al., 2019; Tsoukos et al., 2016), but there is a limited number of studies examining the effects of ROM on fatigue during repeated sets of resistance exercise (Drinkwater et al., 2007; Walker et al., 2012). A recent study compared isometric force production and muscle activation in the bench press exercise performed either at a full ROM or at partial ROM close to elbow extension (Mendonça et al., 2021). Exercise with full ROM resulted in higher muscle activation, sEMG and isometric fatigue, compared with partial ROM, but isometric force was measured 10 min after and not during exercise (Mendonça et al., 2021). Thus, there is limited information regarding the changes in the applied force during fatiguing exercise, and the few studies are limited by indirect measurements of performance, such as the use of a linear position encoder, i.e., kinematic data. (Drinkwater et al., 2007, 2012).

sEMG studies have presented conflicting results regarding the influence of ROM on muscle activation (Da Silva et al., 2017; Gorsuch et al., 2013; Mendonça et al., 2021; Paoli et al., 2010). Mendonça et al. (2021) have shown that sEMG during the bench press exercise is higher in two portions of the pectoralis major muscle and lower in the anterior deltoid muscle when full was compared with partial ROM. However, the load was not adjusted individually in that study for the partial ROM, and arbitrary 130% of 1-RM in the full ROM trial was used. Standardizing the load according to the specific ROM performance is important, and this was shown for the back squat exercise, where sEMG was higher in the partial ROM (close to knee extension) compared to the full ROM (Da Silva et al., 2017). In contrast, another study showed that the displacement of the load during exercise has to be taken into account, since in the seated military press exercise, the full ROM presented the highest sEMG activity in all muscles involved compared with the shorter displacement of the partial ROM (Paoli et al., 2010). Based on this finding, those authors suggested that when the primary aim is the development of strength, then partial ROMs should be avoided. Similarly, Gorsuch et al. (2013) proposed that parallel squats were preferable to lower depth partial squats for safer training and injury prevention in uphill runners, since lower loads were used through a larger ROM, while the sEMG of the rectus femoris and erector spinae activity were higher (Gorsuch et al., 2013). As with force measurements, the effects of fatigue during repeated sets of resistance exercise on sEMG activity have not been examined. Importantly, changes in variables such as the root mean square (RMS) and the median frequency of sEMG which are related to fatigue (Tsoukos et al., 2021) may offer useful information when measured along with changes in force and power.

Thus, examining the effects of full and partial ROM on force generation, fatigue and sEMG would provide useful information for practitioners working with different partial and full ROM movements. Particularly, the lack of comparison between different parts of ROM, e.g., close to the start and close to the end of a movement observed in the literature, makes the present study unique in its aims and methodology. The main aim of the present study was to compare the applied force characteristics (peak force, mean force) and force derivatives (mean and peak power) in conjunction with sEMG data, among three different ROMs (full, top, bottom) in the bench press exercise, against a commonly used load of 65% 1-RM.

## Methods

### Participants

Ten recreationally active men took part in the study (age: 23.2 ± 5.1 years, body height: 1.81 ± 0.07 m, body mass: 81.7 ± 10.1 kg, body fat: 10.3 ± 3.9%). Participants were involved in strength and power sports for at least three years and fulfilled the following criteria: (a) engaged with the bench press exercise for at least three years, (b) able to lift at least 105% of their body weight in the bench press, (c) not using any nutritional supplements or drugs, and (d) no musculoskeletal injuries for at least one year prior to the study. After a detailed explanation of the experimental protocol, the possible risks involved and the right to cease participation at will, a signed written informed consent form was obtained from each participant. The study was approved by the Ethics Committee of the School of P.E. and Sport Science, National and Kapodistrian University of Athens, Greece (protocol code: 1347; protocol date: 24 January 2021) and all procedures were in accordance with the Code of Ethics of the World Medical Association (Helsinki declaration of 1964, as revised in 2013).

### Measures

#### General and Specific Warm-Up

Participants performed a standardized warm-up, before each preliminary and main session, which consisted of five minutes of light cycling on a stationary cycle ergometer (50–60 watt) and five minutes of dynamic stretching of the arm and chest muscles (Tsoukos et al., 2019, 2021). Subsequently, participants also completed a specific warm-up. During the specific warm-up, participants performed eight reps at 50% of the predicted 1-RM, five reps at 75% of the predicted 1-RM and three reps at 90% of the predicted 1-RM. During the specific warm-up and before the main trials, participants completed one set of eight repetitions with 50% of the load that followed (65% of 1-RM) and three minutes later, one set of five repetitions with 75% of the load that followed with a 3-min rest interval in between (Tsoukos et al., 2021; Tsoukos and Bogdanis, 2023).

#### Mean and Peak Force

Ground reaction force was recorded in every set and repetition using two dual-axis force plates (PS-2142; PASCO Scientific, Roseville, CA) which were connected and synchronized to a PC via an interface (SPARKlink Air; PS-2011; PASCO Scientific, Roseville, CA) at a sampling frequency of 1000 Hz and filtered into a customized recording template (PASCO Capstone software v. 2.6; PASCO Scientifics) (Travis et al., 2022). The two force plates were positioned under the front and back mounts of the bench. The total force output from the two dual-axis force plates was calculated with the equation:

F_total_ = F_front_ force plate + F_back_ force plate (van den Tillaar et al., 2012)

Data were filtered using a fourth order reverse Butterworth low-pass digital filter with a cutoff frequency of 25 Hz and customized software (Bogdanis et al., 2018).

Peak force during the concentric phase (ascending of the barbell) was determined from the force-time curves. Peak force was calculated as the highest 10 ms average of the force-time curve (Tsoukos et al., 2018). Peak force was also calculated as a percentage of the barbell’s weight. Mean force was determined as the average of force during all sets and during the initial and final repetitions of each set. Impulse was calculated as the area under the force curve of the vertical ground reaction force (Bogdanis et al., 2014). Mean and peak power were calculated as the product of force and velocity. Velocity was measured by a linear position transducer (Tendo Power Analyzer System v. 314, TENDO Sports Machines, Trencin, Slovak Republic). To reduce bias in comparisons of the measured variables at different parts of each set (i.e., initial and final repetitions) among the three different ROMs, values were averaged for equal time duration at the start and the end of each set (initial and final repetitions). Preliminary measurements showed that the number of repetitions required to equalize the time under tension under the three conditions was three repetitions for the TOP and BOTTOM ROM and two repetitions for the FULL ROM. This procedure has been presented in detail elsewhere and results in a time under tension of around 2.2 s in the initial repetitions (Tsoukos et al., 2024).

#### Surface Electromyographic (sEMG) Activity

sEMG activity of the pectoralis major (PM-pars sternocostalis), anterior deltoid (AD) and triceps brachii (TB-lateral head) muscles of the dominant-right side of the body (Krzysztofik et al., 2021) was analyzed and recorded by a data acquisition unit and dedicated software (Biopac MP35 and Acqknowledge 4.2.0, Biopac Systems Inc., Santa Barbara, CA). Ag/AgCl electrodes (Bipolar; inter-electrode distance: 20 mm) were attached to the skin according to the recommendations of SENIAM (Hermens et al., 2000). The ground electrode was placed on the clavicle. The skin was shaved, cleaned using alcohol, and rubbed with fine sandpaper to keep the impedance between the two electrodes low. Sampling frequency was set at 2000 Hz for sEMG recordings, amplified (gain = 1000), and the signal was subsequently filtered (band pass = 30–500) (Girard et al., 2018). The Root Mean Square (RMS) was calculated between the onset and the end of the burst. An investigator defined the onset of each burst manually. RMS values (peak value) of the three examined muscles were normalized to the peak sEMG amplitude obtained during two maximum voluntary isometric contractions (MVICs) at two different elbow angles, performed after the specific warm-up under each condition (elbow angles: 91.6 ± 3.9^°^ and 141.1 ± 6.4^°^). Participants performed four 3-s repetitions of isometric bench press exercise, separated by a 3-min rest interval (Tsoukos et al., 2021). The average of the two highest values for the same elbow angle were kept for further analyses and the values of all sEMG variables were expressed as a percentage of MVIC (%MVIC).

Median frequency of the EMG power spectrum and integrated sEMG (iEMG) activity (i.e., area under the sEMG curve) were calculated and expressed as a percentage of the peak sEMG activity obtained during the MVICs trials of the examined muscles. Median frequency (MF) was calculated for the initial and the last part of each set as an index of muscle fatigue. MF is expressed as a percentage of that measured during MVIC. The integrated sEMG activity (iEMG-area under the sEMG curve) for all agonist muscles was assessed as a total and also for the initial and final repetitions in all sets under each condition. The iEMG was presented as a percentage of the peak sEMG as previously described (Tsoukos et al., 2021).

### Design and Procedures

A repeated measures design was used to compare the effects of different ranges of motion on the applied force, power and sEMG variables during three sets to instant exhaustion with maximum intended velocity against a load of 65% of the 1-RM in the bench press exercise. Participants were fully familiarized with the experimental procedures and protocol during three preliminary sessions, in which the ROM-specific 1-RM was measured (Tsoukos et al., 2019, 2021; Tsoukos and Bogdanis, 2023). Thereafter, three main trials were performed 5–7 days apart. Each trial consisted of three sets to instant exhaustion with a 2-min rest interval between sets on a Smith machine performed as fast as possible (movement tempo X:0:X:0), against a moderate load of 65% of the 1-RM with three different ROMs: (a) full ROM (FULL, displacement: 42.7 ± 3.4 cm), (b) partial ROM in which the barbell moved at the bottom half of the bench press (BOTTOM; displacement: 25.5 ± 2.3 cm), and (c) partial ROM in which the barbell moved at the top half of the bench press (TOP; displacement: 24.5 ± 2.3 cm). The TOP ROM was set using block foam pads wrapped with tapes (total thickness approx. half of the individual full ROM) placed on the chest of participants. For the BOTTOM ROM, participants were guided by one spotter, who placed their hand near the vertical rail at half distance of the individual FULL ROM from the participants’ chest. Each participant had to touch the bar on the spotter’s hand in every repetition. During all ROM conditions, participants were asked to slightly touch the barbell on their chest or the foam pad.

The main dependent variables for the three different ROMs were: mean and peak force, mean and peak power, and sEMG variables of the pectoralis major, triceps brachii and anterior deltoid muscles (i.e., sEMG Root Mean Square (RMS), integrated sEMG (iEMG) and median frequency (MF) of the EMG power spectrum). Changes in the number of repetitions, mean and peak barbell velocity, time under tension and fatigue have been published elsewhere (Tsoukos et al., 2024).

### Statistical Analysis

Statistical analyses were conducted using the SPSS Statistics Ver. 23 (IBM Corporation, USA). All data are presented as means and standard deviations (SD). Normality, homogeneity, and sphericity of the data were verified by the Shapiro-Wilk, Levene’s, and Mauchly’s tests, respectively. The comparison of variables during three sets to failure as a total, were examined by one-way repeated measures analysis of variance (ANOVA) to detect any differences among the three different ROMs. This was performed to investigate any differences among the three ROMs of the total exercise. Three-way repeated measures ANOVA (3 ROMs [FULL, TOP, BOTTOM] x 3 sets [1^st^, 2^nd^, 3^rd^] x 2 parts [initial and last]) was conducted to examine differences among the three ROMs, sets and parts. When appropriate, a two-way ANOVA was used. A Tukey’s post-hoc test was performed when a significant main effect or interaction was observed. The effect sizes for main effects and interactions were determined by Partial eta squared (η^2^_p_) values, which were classified as small (0.01 to 0.059), moderate (0.06 to 0.137) and large (>0.137). For pairwise comparisons, the effect size was determined by Hedges’ g (small, <0.3; medium, 0.3–0.8; large, >0.8). Statistical significance was set at *p* < 0.05.

## Results

### Mean Force and Impulse

The three-way ANOVA (ROM x SET x PART) for mean force showed no interactions (*p* > 0.42). However, there were main effects of ROM (*p* < 0.001; η^2^_p_ = 0.86) and PART (*p* = 0.013; η^2^_p_ = 0.51). Tukey’s post-hoc tests revealed that mean force was significantly higher during TOP compared to FULL (817 ± 80 vs. 657 ± 98 N, respectively, *p* < 0.001; Hedges' g = 1.7) and BOTTOM (623 ± 122 N, *p* < 0.001; Hedges' g = 1.8). No differences were observed between FULL and BOTTOM (*p* > 0.05). Tukey’s post-hoc tests also showed a significant, but small decrease (2%) in mean force in the final compared to the initial repetitions irrespective of ROM (*p* = 0.013).

A higher number of repetitions was performed in TOP (55.2 ± 9.8) and BOTTOM (49.1 ± 16.5), compared to FULL ROM (32.2 ± 6.5) (TOP from FULL: *p* < 0.01; Hedges' g = 2.6; BOTTOM from FULL: *p* < 0.01; Hedges' g = 1.3). The three-way ANOVA (ROM x SET x PART) for impulse showed a ROM x PART interaction (*p* = 0.45), and main effects of ROM (*p* < 0.001; η^2^_p_ = 0.59) and PART (*p* = 0.001; η^2^_p_ = 0.95). Tukey’s post-hoc tests revealed that impulse was significantly higher during TOP compared to FULL (49.02 ± 10.21 vs. 34.48 ± 7.89 kN·s, respectively, *p* < 0.001; Hedges' g = 1.5) and BOTTOM (30.60 ± 9.06 kN·s, *p* < 0.001; Hedges' g = 1.8). No differences were observed between FULL and BOTTOM (*p* > 0.05). Tukey’s post-hoc tests also showed that participants generated higher impulse in the initial compared to the final repetitions in all ROMs (*p* < 0.001).

#### Peak Force

**Figure 1:**
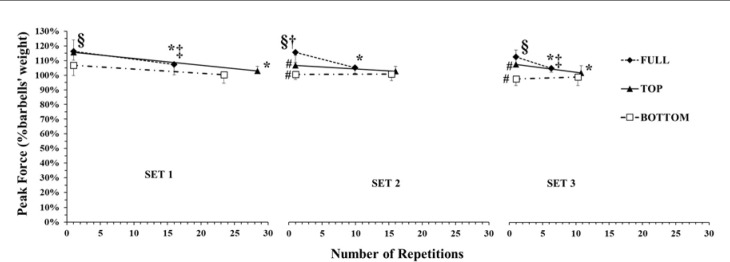
Peak force expressed as a percentage of the barbell’s weight during the different ROMs in the three sets. *: *p <* 0.05 from the initial part in the corresponding SET and ROM; #: *p <* 0.05 from set 1 in the corresponding ROM and part; †: *p <* 0.05 from TOP in the corresponding set and part; §: *p <* 0.05 FULL and TOP from BOTTOM in the corresponding set and part; §: *p <* 0.05 from BOTTOM in the corresponding set and part; ‡: *p <* 0.05 FULL from BOTTOM in the corresponding set and part

The ANOVA showed a three-way interaction (*p* < 0.01; η^2^_p_ = 0.33) for peak force. Peak force at the start of set 1 was different among ROMs, being higher in TOP (943 ± 83 N) compared with FULL (774 ± 98 N) and BOTTOM (683 ± 101 N, all differences *p* < 0.01). Tukey’s post-hoc tests revealed a 6.7 to 8.8% decrease in peak force from the initial to the final repetitions of each set in FULL ROM (*p* < 0.01, Figure 1). In TOP ROM, peak force decreased from the initial to the final repetitions in sets 1 and 3 (by 10.6 and 5.2%, respectively, *p* < 0.01), but remained unchanged in set 2. Peak force at the start of sets 2 and 3 did not recover to the set 1 level and remained about 7% lower. In BOTTOM ROM, there was no change in peak force within or between sets (Figure 1).

#### Mean Power

**Figure 2:**
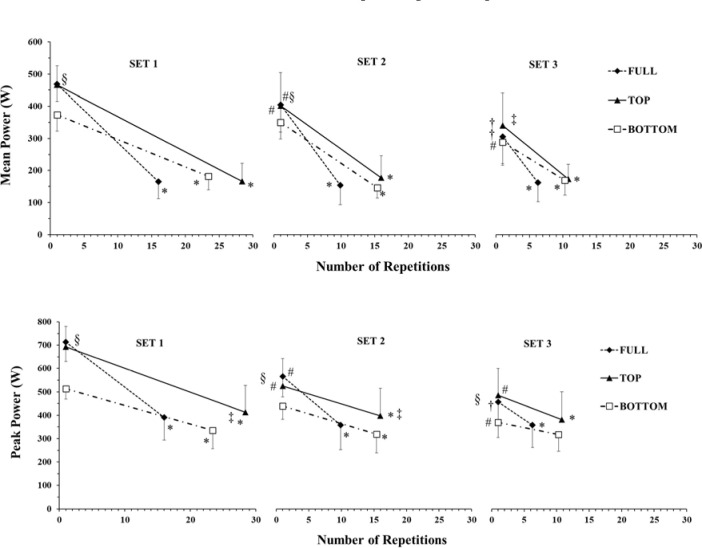
Mean and peak power during the different ROM in the three sets. * *p <* 0.01 from the initial part of all ROMs in the corresponding set; #: *p <* 0.01 from set 1 in the corresponding ROM and part; †: *p <* 0.01 from set 1 and set 2 in the corresponding ROM and part; §: *p <* 0.05 TOP and FULL from BOTTOM in the corresponding set and part; ‡: *p <* 0.05 TOP from BOTTOM in the corresponding set and part

The ANOVA for mean power revealed a significant three-way interaction (ROM x SET x PART; *p* < 0.01; η^2^_p_ = 0.34). Mean power at the start of set 1 was similar in FULL and TOP (486 ± 53 and 486 ± 59 W, respectively) and higher than in BOTTOM (373 ± 50 W, *p* < 0.001, Hedges' g = 1.8 and 1.6). Mean power at the start of set 2 was also similar in FULL and TOP and higher than in BOTTOM (*p* < 0.05; Hedges' g = 0.8 and 0.6), while in set 3 mean power was higher in TOP compared to BOTTOM (*p* < 0.05; Hedges' g = 0.6, Figure 2). Tukey’s post-hoc tests showed that mean power decreased to the same level in the final repetitions in all ROMs and sets (*p* < 0.001) (Figure 2). The percent decrease in mean power was similar in all ROMs (55.5 to 64.7%), but it dropped much faster in terms of the number of repetitions in FULL compared with TOP and BOTTOM (Figure 2). Between set comparisons showed that mean power in the initial repetitions progressively decreased from set 1 to set 2 and to set 3 (by 13.5 and 28.1%, respectively, *p* < 0.01).

#### Peak Power

The ANOVA for peak power revealed a significant three-way interaction (ROM x SET x PART; *p* < 0.047; η^2^_p_ = 0.23). Peak power at the start of set 1 was similar in FULL and TOP (714 ± 83 and 692 ± 88 W, respectively) and higher than in BOTTOM (514 ± 45 W, *p* < 0.001, Hedges' g = 2.9 and 2.4). Peak power at the start of set 2 and set 3 was also similar in FULL and TOP and higher than in BOTTOM (*p* < 0.05; Hedges' g = 1.0 and 0.72, Figure 2). Tukey’s post-hoc tests showed that peak power decreased to the same level in the final repetitions in all ROMs and sets (*p* < 0.001) except for the 3^rd^ set in BOTTOM, where it remained at very low values (Figure 2). The percentage decrease in peak power was similar in all ROMs (29.4 to 45.3%), but it dropped much faster in terms of the number of repetitions in FULL compared with TOP and BOTTOM (Figure 2). Between set comparisons showed that peak power in the initial repetitions progressively decreased from set 1 to set 2 and to set 3 (by 13.5 and 28.1%, respectively, *p* < 0.01).

#### sEMG Activity (RMS)

**Figure 3:**
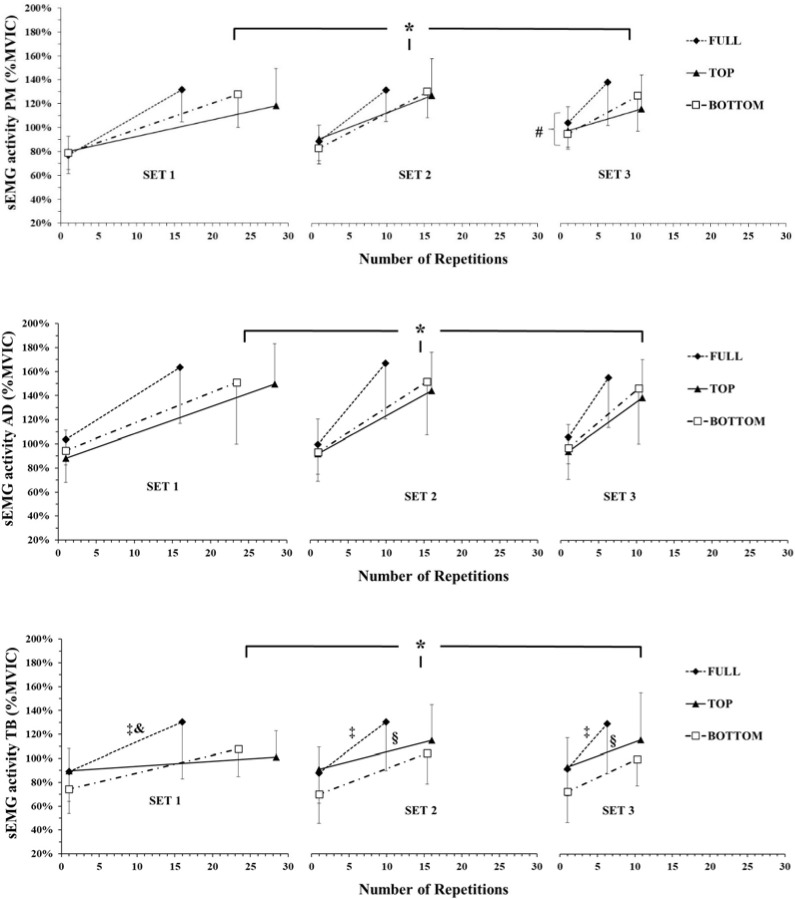
sEMG activity (RMS) of pectoralis major (PM), anterior deltoid (AD) and triceps brachii (TB) muscles expressed as a percentage of MVIC (%MVIC). PM muscle showed a set x part interaction. AD muscle showed a main effect for part only. TB showed a ROM x set interaction and a ROM x part interaction. *: *p <* 0.01 from the initial part in the corresponding set during all ROMs; #: *p <* 0.05 from set 1 in all ROMs; ‡: *p <* 0.01 during FULL (initial and last parts simultaneously) from BOTTOM; §: *p <* 0.01 during TOP (initial and last parts simultaneously) from BOTTOM; &: *p <* 0.01 during FULL (initial and last parts simultaneously) from TOP.

The three-way ANOVA did not show a significant ROM x SET x PART interaction for sEMG of the PM muscle (*p* = 0.91; η^2^_p_ = 0.03). However, there was a two-way interaction (SET x PART, *p* = 0.01; η^2^_p_ = 0.35). Post-hoc comparisons showed that sEMG of the PM muscle was higher in the last part compared to the initial part in all sets and ROMs (*p* < 0.001) (Figure 3). Also, sEMG of the PM muscle was higher in the initial part of the 1^st^ set compared to the 3^rd^ set in all ROMs simultaneously (*p* < 0.01; Hedges' g = 1.2).

The three-way ANOVA did not show a significant ROM x SET x PART interaction for the TB (*p* = 0.31; η^2^_p_ = 0.12). However, the following two-way interactions were found: ROM x SET (*p* = 0.03; η^2^_p_ = 0.25) and ROM x PART (*p* = 0.001; η^2^_p_ = 0.41). Post-hoc comparisons (ROM x SET interaction) showed that sEMG of the TB did not change from set to set during the different ROMs (*p* > 0.05). sEMG of the TB was higher in FULL compared to TOP in both parts, during set 1 only (*p* < 0.01; Hedges' g = 0.4) and compared to BOTTOM in both parts during set 1 (*p* < 0.01; Hedges' g = 0.5), set 2 (*p* < 0.01; Hedges' g = 0.6) and set 3 (*p* < 0.01; Hedges' g = 0.7) (Figure 3). Post-hoc comparisons (ROM x PART interaction) showed that sEMG of the TB was higher at the initial repetitions compared to the final repetitions during all ROMs in all sets simultaneously (*p* < 0.01). sEMG of the TB was higher in FULL compared to TOP at the final repetitions in all sets simultaneously (*p* < 0.01; Hedges' g = 0.5) and compared to BOTTOM at the initial (*p* < 0.01; Hedges' g = 0.7) and final repetitions in all sets simultaneously (*p* < 0.01; Hedges' g = 0.74). Furthermore, sEMG of the TB was higher in TOP compared to BOTTOM at the initial repetitions irrespective of the set (*p* < 0.01).

The three-way ANOVA did not show a significant ROM x SET x PART interaction for the AD (*p* = 0.44; η^2^_p_ = 0.10). However, iEMG showed a main effect for PART (*p* < 0.001; η^2^_p_ = 0.86). Tukey’s post hoc tests revealed that the sEMG of the AD muscle was higher at the final compared to the initial repetitions (*p* < 0.01; Hedges' g = 1.6) (Figure 3).

#### Integrated sEMG

**Figure 4:**
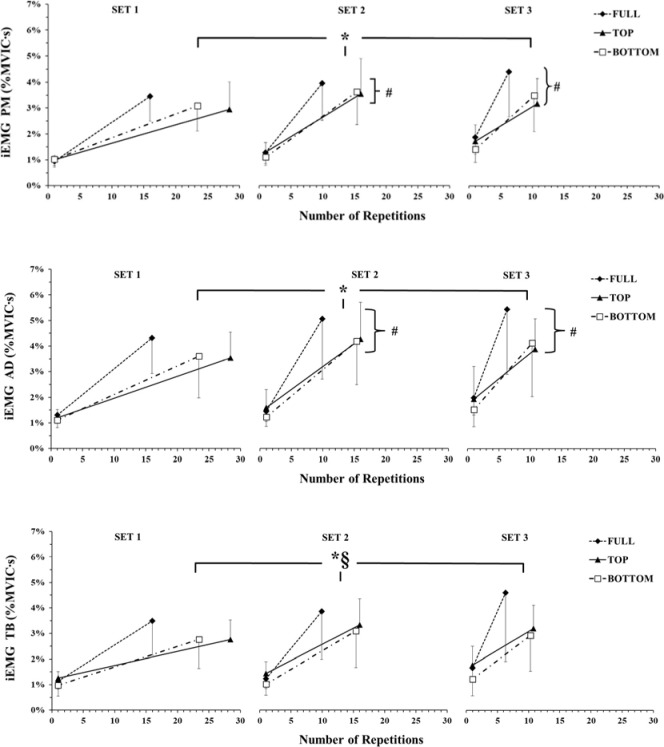
Integrated sEMG (iEMG) of pectoralis major (PM), anterior deltoid (AD) and triceps brachii (TB) muscles expressed as a percentage of MVIC (%MVIC). PM and AD muscles showed main effects for set and part. TB showed a ROM x part interaction. *: *p <* 0.01 from the initial part during all ROMs; #: *p <* 0.01 from set 1 during all ROMs and parts; §: *p <* 0.05 FULL compared to BOTTOM at the final repetitions irrespective of set.

Integrated sEMG (iEMG) of the PM, AD and TB muscles for each ROM for each part (initial and last) are presented in Figure 4. The three-way ANOVA did not show a significant ROM x SET x PART interaction for iEMG of the PM muscle (*p* = 0.66; η^2^_p_ = 0.06). However, a main effect was found for SET (*p* < 0.01; η^2^_p_ = 0.86) and PART (*p* < 0.01; η^2^_p_ = 0.86). Tukey’s post hoc tests (main effect SET) revealed that iEMG of the PM muscle was higher during set 1 compared to set 2 (*p* < 0.01; Hedges' g = 0.3) and set 3 (*p* < 0.01; Hedges' g = 0.4). Also, Tukey’s post hoc tests (main effect for PART) revealed that iEMG of the PM muscle was higher in the final compared to the initial repetitions (*p* < 0.01; Hedges' g = 2.2).

The three-way ANOVA did not show a significant ROM x SET x PART interaction for iEMG of the TB muscle (*p* = 0.56; η^2^_p_ = 0.08). However, there was a two-way interaction (ROM x PART, *p* = 0.049; η^2^_p_ = 0.28). Tukey’s post hoc tests showed that iEMG of the TB was higher at the initial compared to the final repetitions during all ROMs (*p* < 0.01). iEMG of the TB muscle was higher in FULL compared to BOTTOM at the final repetitions (*p* = 0.018; Hedges' g = 0.6). FULL showed also a trend for significance compared to the final repetitions during TOP (*p* = 0.056; Hedges' g = 0.5).

The three-way ANOVA did not show a significant ROM x SET x PART interaction for iEMG of the AD muscle (*p* = 0.68; η^2^_p_ = 0.06). However, there was a main effect for SET (*p* < 0.01; η^2^_p_ = 0.58) and PART (*p* < 0.01; η^2^_p_ = 0.88). Tukey’s post hoc tests (main effect for SET) revealed that the iEMG of the AD muscle was higher during set 1 compared to set 2 (*p* < 0.01; Hedges' g = 0.24) and set 3 (*p* < 0.01; Hedges' g = 0.33). Also, Tukey’s post hoc tests (main effect for PART) revealed that the iEMG of the AD muscle was higher at the final compared to the initial repetitions (*p* < 0.01; Hedges' g = 2.0).

#### Median Frequency

The three-way ANOVA did not show a significant ROM x SET x PART interaction for MF of the PM muscle (*p* = 0.06; η^2^_p_ = 0.21). However, a two-way interaction (SET x PART) was observed (*p* < 0.01; η^2^_p_ = 0.70). Post-hoc comparisons showed that MF of the PM muscle was lower in the last compared to the initial part in all sets (*p* < 0.001). Also, MF of the PM muscle was higher in the initial part of the 1^st^ set compared to the 2^nd^ (*p* < 0.01; Hedges' g = 0.82) and 3^rd^ sets (*p* < 0.01; Hedges' g=1.03), respectively.

The three-way ANOVA showed a three-way interaction for MF of the TB muscle (*p* = 0.032; η^2^_p_ = 0.25). Tukey’s post-hoc tests revealed that MF of the TB muscle was always higher in the initial compared to the final repetitions at all ROMs (*p* < 0.001). MF of the TB muscle was higher during FULL compared to BOTTOM at the initial repetitions of the 1^st^ set (*p* = 0.046; Hedges' g = 0.5) and during TOP compared to BOTTOM at the initial repetitions of the 2^nd^ set (*p* < 0.01; Hedges' g = 0.61).

The three-way ANOVA did not show a significant ROM x SET x PART interaction for MF of the AD muscle (*p* = 0.12; η^2^_p_ = 0.18). However, the following two-way interactions were observed: COND x PART (*p* < 0.05; η^2^_p_ = 0.31) and SET x PART (*p* < 0.01; η^2^_p_ = 0.51). Post-hoc comparisons showed that MF of the AD muscle was lower in the last compared to the initial part in all sets and ROMs (*p* < 0.001). Also, MF of the AD muscle was higher during TOP in the initial part of all sets compared to BOTTOM (*p* < 0.01; Hedges' g = 0.6). We also found a higher decrease in the last part of set 1 compared to set 3 irrespective of ROM (*p* < 0.01; Hedges' g = 0.5).

## Discussion

This study compared the applied force characteristics and surface electromyographic activity (sEMG) of prime mover muscles during three sets to failure in the bench press exercise, performed at three different ranges of motion (FULL, TOP and BOTTOM) with a load at 65% of 1-RM with maximum intended velocity. The main findings were that when exercising at the TOP ROM, participants applied a higher mean and peak force and produced a higher impulse than under FULL and BOTTOM conditions, with the latter demonstrating the lowest values. Mean force decreased by only 2% from the initial to the final repetitions in all sets and ROMs, while peak force also showed small decreases from the initial to the final repetitions of each set in FULL ROM (by 6.7 to 8.8%), while at the TOP ROM these decreases were smaller and in the BOTTOM ROM non-existent. However, within each set there was a large decrease in peak (by 29.4 to 45.3 %) and mean power (by 55.5 to 64.7%), which indicates that these decreases are dependent on velocity loss rather than force decrements. sEMG variables (RMS, iEMG) increased, and MF decreased at the end of each set in all ROMs. However, sEMG variables of the TB muscle showed different responses among ROMs, with RMS and iEMG being higher in FULL and TOP and lower in BOTTOM (FULL>TOP>BOTTOM). Also, MF in TB and AD muscles was higher during TOP and FULL compared with BOTTOM at the initial repetitions. Fatigue was evident in all variables with mean and peak force as well as mean and peak power progressively decreasing in the last compared with the initial repetitions. However, peak force and peak power decreased much less compared to mean power.

One main finding of the present study was that mean and peak force was higher in TOP compared to FULL and BOTTOM. This result is due to a combination of more favorable musculoskeletal mechanics and force-muscle length relationship in this ROM. When bench pressing with wider joint angles there are lower resistive torques and muscles operate close to their optimum length, resulting in higher force generation (Bloomquist et al., 2013; Bogdanis et al., 2019; Murphy et al., 1996). A previous study showed differences in applied force between different ROMs in the bench press exercise, but this was in isometric muscle actions (Murphy et al., 1996). Those authors found that maximal isometric force was almost 30% higher at an elbow angle of 120^°^ which is close to the starting angles in the present study (TOP: 114 ± 4^°^) compared to an angle of 90^°^ (Murphy et al., 1996) which is also close to the starting angles of the FULL and BOTTOM ROMs in the present study (BOTTOM: 77 ± 9^°^ and FULL: 78 ± 12^°^) (Tsoukos et al., 2024). Interestingly, the percentage difference in force among TOP, FULL and BOTTOM ROM in that study were similar to the values reported in the present study (Murphy et al., 1996). Also, impulse was higher in TOP compared to FULL and BOTTOM ROMs. Impulse is the integral of force with respect to time and, as force and time under tension were significantly higher in TOP compared to FULL and BOTTOM, it is reasonable for impulse to be significantly higher in TOP compared to FULL and BOTTOM ROMs (Tsoukos et al., 2024). Thus, it may be suggested that the TOP ROM may be used to increase absolute loading in the bench press exercise and help athletes in sports where the absolute maximum force of elbow extensors is essential, i.e., individuals whose sports involve forceful pushing or throwing actions, such as the shot put or boxing (Lopez-Laval et al., 2020; Terzis et al., 2003).

Mean and peak power output was higher in TOP and FULL than in BOTTOM ROM. Power is defined as the product of force and velocity, and thus the higher power is a result of both higher force in FULL compared to BOTTOM (Figure 2), and higher velocities in FULL than BOTTOM (Tsoukos et al., 2024). This finding may be related to a combination of a higher force production and sEMG activity of the TB muscle in TOP (Figures 2 and 3) compared to BOTTOM and because of the existence of the sticking region (i.e., mechanical disadvantage during the initial phase of the lift) during BOTTOM ROM (Martínez-Cava et al., 2019) which has been shown to present significantly lower power values (Drinkwater et al., 2007). In a previous study, it was shown that mean and peak velocity when bench-pressing at BOTTOM ROM was higher than TOP because of the significantly lower absolute load used (Tsoukos et al., 2024), and it could be speculated that power production would have been equal in the two partial ROMs, as a lower force was accompanied by higher velocity.

Fatigue has been defined as a reduction in the required force and/or power production during repeated muscle actions (Bogdanis, 2012; Enoka and Duchateau, 2008). Within each set there was a large decrease in peak (by 29.4 to 45.3%) and mean power (by 55.5 to 64.7%). However, we observed very small decrements within each set, in mean force (by only 2%) and peak force (from 8.8% to zero). In a companion paper (Tsoukos et al., 2024), we found large decreases in peak barbell velocity (by 28–44%) and mean barbell velocity (70–80%) from the initial to the final repetitions of each set, which in conjunction with the present data, show that the decreases in mean and peak power are due to a large drop in velocity and much less due to a decrease in force. The manifestation of fatigue as a larger decrease in velocity than force of movement has been previously shown for other types of maximal exercise, such as repeated sprinting (Bogdanis et al., 2007). In that study, peak power output decline was more evident when participants performed all-out sprints with a higher pedal rate and thus, velocity of muscle contraction (Bogdanis et al., 2007). Interestingly, in the present study, peak and mean power decrease was faster in FULL, where the velocity of movement was also higher, yet it was much lower in TOP, where velocity of movement was lower (Tsoukos et al., 2024), despite the fact that mean and peak force were higher in TOP. The fact that peak and mean force showed little or no changes in set 3 for BOTTOM, together with the significant decrease in power output suggest that fatigue depends more on velocity loss than force loss. Thus, the relatively lower overall performance in BOTTOM, although force was lower, may be related to the faster decline in movement velocity (Tsoukos et al., 2024). Previous research has shown that fatigue is higher at longer muscle lengths, i.e., BOTTOM ROM, and this may be the case in the present study (Lee et al., 2007; Tsoukos et al., 2016).

sEMG increased and MF decreased from the initial to the final repetitions. This is in line with previous studies which have shown that during fatigue sEMG of the agonist muscles increases and MF decreases (Stastny et al., 2017; Tsoukos et al., 2021). The higher sEMG activity in the TB muscle during FULL compared to BOTTOM may be related to the higher mean and peak velocity that participants achieved (Tsoukos et al., 2024). When training in a larger ROM, mean and peak velocity of movement are higher compared with a partial ROM (Drinkwater et al., 2012; Krzysztofik et al., 2020) and sEMG activity is influenced by the movement velocity (Calatayud et al., 2018; Jakobsen et al., 2013; Tsoukos et al., 2021). A recent study showed that in FULL ROM, mean and peak barbell velocities were higher than in BOTTOM ROM (Tsoukos et al., 2024). However, these results were evident in the beginning of the first set (Tsoukos et al., 2024) and the differences in the sEMG values in the present study are observed in all sets. This finding may be related to the higher peak force values that participants achieved in FULL and TOP ROM compared to BOTTOM ROM in the present study, while it is well known that sEMG is related to the level of force production (Disselhorst-Klug et al., 2009).

**Figure 5:**
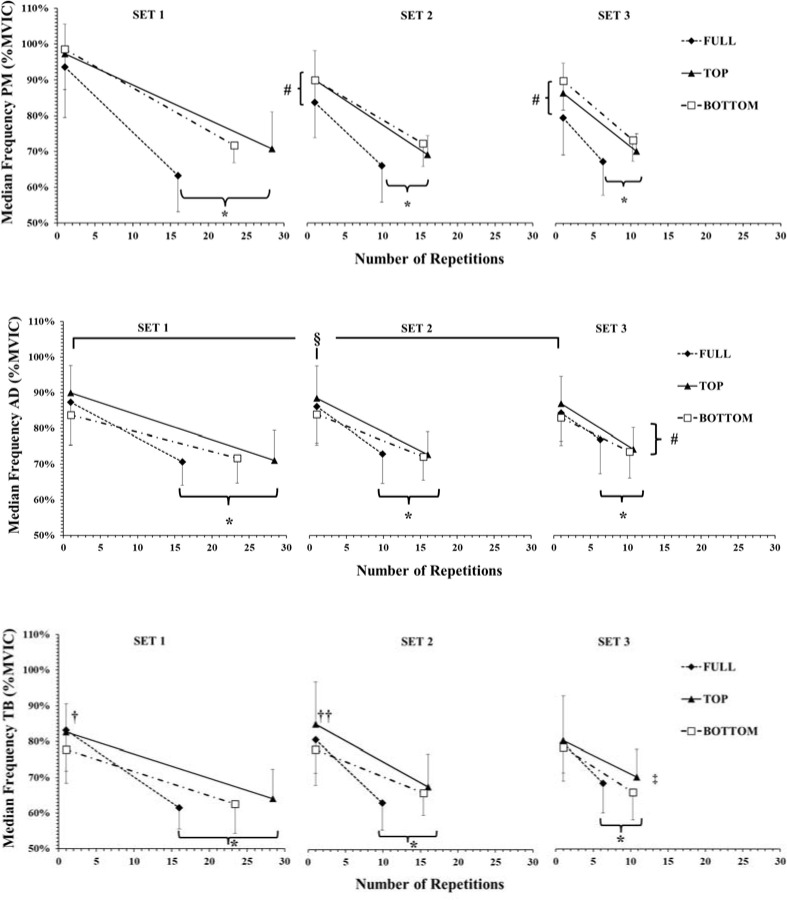
Median frequency of the EMG signal of pectoralis major (PM), anterior deltoid (AD) and triceps brachii (TB) muscles expressed as a percentage of MVIC (%MVIC). PM muscle showed a set x part interaction. AD muscle showed ROM x part and set x part interactions. TB showed a ROM x set x part interaction. *: *p <* 0.01 from the initial part during all ROMs; #: *p <* 0.05 from set 1 in all ROMs in the corresponding part; ‡: from set 1 in TOP and FULL ROMs in the corresponding part; §: *p <* 0.05 TOP compared to BOTTOM irrespective of set; †: *p <* 0.05 FULL from BOTTOM in the corresponding set and part; ††: *p <* 0.01 TOP from BOTTOM in the corresponding set and part.

In the present study, MF of the AD and TB muscles was higher in TOP compared to BOTTOM ROM at the initial repetitions (Figure 5). Research has shown that MF is torque or force dependent (Miller and Croce, 2002). Thus, the higher the force the higher the MF. This explanation may also be valid for the higher MF during the initial repetitions of the FULL ROM compared to BOTTOM in the 1^st^ set of TB muscle. Higher initial MF values have been associated with faster muscle fiber conduction velocity as well as with higher recruitment of fast twitch fibers (Kupa et al., 1995).

## Conclusions

The present study showed that when training at the TOP ROM, participants applied higher mean and peak force and higher impulse than under FULL and BOTTOM conditions, possibly due to a combination of more favorable musculoskeletal mechanics and a force-muscle length relationship. A large decrease was found in peak (by 29.4 to 45.3 %) and mean power (by 55.5 to 64.7%) within each set. However, the decrements in mean and peak force were very small and thus power decrements may be attributed to velocity loss rather than force loss. Changes in sEMG reflected the decrements in power output. Fatigue was accompanied by an increase in RMS and iEMG, and a decrease in MF in all muscles, with triceps brachialis sEMG reflecting to a greater extent the force and power differences. The dependence of fatigue on velocity rather than force loss during bench press exercise at different ROMs has significant practical implications for training athletes and healthy individuals.
